# Prevalence and Antimicrobial Resistance of *Listeria monocytogenes* in Different Raw Food from Reynosa, Tamaulipas, Mexico

**DOI:** 10.3390/foods13111656

**Published:** 2024-05-25

**Authors:** Paulina Guel-García, Francisco Javier García De León, Guadalupe Aguilera-Arreola, Antonio Mandujano, Maribel Mireles-Martínez, Amanda Oliva-Hernández, María Antonia Cruz-Hernández, Jose Vasquez-Villanueva, Gildardo Rivera, Virgilio Bocanegra-García, Ana Verónica Martínez-Vázquez

**Affiliations:** 1 Centro de Biotecnología Genómica, Instituto Politécnico Nacional, Reynosa C.P. 88710, Tamaulipas, Mexico; gguelg1700@alumno.ipn.mx (P.G.-G.); jamh_93@hotmail.com (A.M.); mmireles@ipn.mx (M.M.-M.); aoliva@ipn.mx (A.O.-H.); macruzh@ipn.mx (M.A.C.-H.); giriveras@ipn.mx (G.R.); vbocanegra@ipn.mx (V.B.-G.); 2 Laboratorio de Genética para la Conservación, Centro de Investigaciones Biológicas del Noroeste, S.C., La Paz C.P. 23090, Baja California Sur, Mexico; fgarciadl@cibnor.mx; 3 Laboratorio de Bacteriología Medica, Escuela Nacional de Ciencias Biológicas, Instituto Politécnico Nacional, México City C.P. 11340, Mexico; lupita_aguilera@hotmail.com; 4 Facultad de Medicina Veterinaria y Zootecnia, Universidad Autónoma de Tamaulipas, Ciudad Victoria C.P. 87274, Tamaulipas, Mexico; jvazquez@docentes.uat.edu.mx

**Keywords:** *Listeria monocytogenes*, serotype, virulence, foodborne bacteria, PCR, antimicrobial resistance

## Abstract

*Listeria* (*L.*) *monocytogenes* is an opportunistic foodborne pathogen that causes listeriosis in humans and animals, reaching up to 30% case mortality. There are only a few reports in Mexico about the *L. monocytogenes* strains found in various foods. The aim of this study was to determine the prevalence of *L. monocytogenes*, serogroups, virulence genes, and antimicrobial resistance in different foods from Reynosa, Tamaulipas, Mexico. *L. monocytogenes* strains were characterized by microbiological and molecular methods. Susceptibility to 12 antibiotics was determined according to CLSI and EUCAST. A total of 300 samples of seafood, pasteurized and raw milk, cheese, beef, and chicken were collected from supermarkets and retail markets. The presence of *L. monocytogenes* was detected in 5.6% of the samples. Most strains belonged to serogroups 4b, 4d, and 4e (68.4%). All strains presented a minimum of four virulence genes; the most common were *actA*, *hly*, and *plcB* (92.1%). A high percentage of antimicrobial susceptibility was observed, with resistance only to STX-TMP (78.9%), STR (26.3%), MEM (21.0%), and E (2.6%). These results show that the foods in Reynosa, Tamaulipas, are a reservoir of *L. monocytogenes* and represent a potential health risk.

## 1. Introduction

*Listeria (L.) monocytogenes* is an opportunistic bacterium that can produce listeriosis in humans and animals, with a significant mortality rate of 20–30% worldwide [1,2]. These bacteria are characterized by their ability to survive and multiply in adverse environmental conditions that allow them to be present in different reservoirs, such as animals, water, soil, surfaces, and food [3].

The Centers for Disease Control and Prevention (CDC) estimates that *Listeria* is the third leading cause of death from foodborne illness in the United States [4]. In that country alone, the CDC estimates, 1600 people become sick from listeriosis each year and about 260 die [4]. The difference between infections with mild and severe symptoms depends on the age of the infected person, their immune status, the number of bacterial cells ingested, and the virulence properties of the strain [2]. As an invasive intracellular pathogen, *L. monocytogenes* depends on an arsenal of virulence factors to facilitate its colonization of the gastrointestinal tract, survival, and spread of infection [5].

*L. monocytogenes* consists of four major evolutionary lineages (I, II, III and rare lineage IV), including 14 recognized serotypes that are grouped into four PCR serogroups [6].

Lineages I and II include most of the isolates linked to clinical cases of listeriosis. The lineage I group contains serotypes 1/2b, 3b, and 4b and has greater pathogenic potential than lineage II, which is composed of serotypes 1/2a, 1/2c, 3a, and 3c. Lineage IV groups serotypes 4a and 4c and an atypical 4b [6,7].

In listeriosis, four pathogenicity islands have been identified; LIPI-1 (*act*A, *prf*A, *hly*A, *mpl*, *plc*A, *plc*B) and LIPI-2 (*inl*A, *inl*B, *inl*C, *inl*J) promote adhesion, invasion, and the spread from cell to cell within the host organism, LIPI-3 (*lls*A, *ll*G, *lls*H, *llsX*, *lls*B, *llsY*, *llsD*, *llsP*) deregulates host activity during infection, and LIPI-4 (*lic*C, *lic*B, *lic*A, *lm* 900558-70012, *lm* 900558-70013 and maltose-60-phosphate-glucosidase (*glvA*)) is strongly related to neural and placental infections [8,9,10].

The standard treatment of severe *L. monocytogenes* infections is based on amoxicillin, aminopenicillin, or ampicillin alone or in combination with an aminoglycoside (frequently gentamicin) [5,11,12]. The treatment may also include penicillin, sulfamethoxazole/trimethoprim, vancomycin, and erythromycin [4,13,14,15]. These choices are based on antimicrobial agents that are not influenced by the natural (intrinsic) resistance of *L. monocytogenes*. Some factors, such as variance in geographical environments and differences in the application of antibiotics, can influence the resistance or susceptibility patterns of *L. monocytogenes* isolates [16]. Several studies have detected strains of *L. monocytogenes* (obtained from food sources) that are resistant to one or several antibiotics, even those used for treatment [17,18,19]. This complicates its treatment and has generated concern in the health sector.

In Mexico, reporting cases of listeriosis is not mandatory, and since there are no epidemiological statistics, the risk it represents to public health is unknown [19,20]. Although the transmission of listeriosis is mainly associated with food, only a few studies have been published on the prevalence or resistance to antibiotics; for example, Cruz-Pulido et al. [19] reported a 1.4% prevalence of *L. monocytogenes* in frozen chicken from Reynosa, Tamaulipas (which shares a border with the United States). Subsequently, Rubio et al. [21] detected *L. monocytogenes* in 27.7% of strains isolated from beef samples obtained from cities in the north, center, and south of the country (Monterrey, Mexico City, and Tabasco). Then, in Jalisco in 2014, Rosas et al. [22] reported *L. monocytogenes* in 17.4% of cheese samples. In recent years, Chávez-Martínez et al. [23] have reported a 2.2% prevalence of *L. monocytogenes* in cheese from Chihuahua. All these studies have shown the presence of *L. monocytogenes* in different foods, with variable prevalence percentages based on geographical area. However, it is important to highlight that although the presence of *L. monocytogenes* in food already represents a potential risk to the consumer’s health, the published information is limited; therefore, this also limits the reaction capacity for the health sector in Mexico.

Therefore, to carry out a more efficient risk assessment and establish control strategies for this bacterium, it is necessary to understand its adaptive mechanisms.

For these reasons, we are interested in evaluating the prevalence levels of *L. monocytogenes* in a border city of the United States and Mexico and defining the serogroups, presence of virulence genes, and antimicrobial resistance in raw food.

## 2. Materials and Methods

### 2.1. Sample Collection

A total of 300 samples were collected from supermarkets and retail markets in Reynosa, Tamaulipas. The samples collected were raw poultry (*n* = 60), ground beef (*n* = 60), seafood (*n* = 20 fish, *n* = 20 shrimp, and *n* = 20 crabs), cheese (*n =* 30 fresh cheese and *n =* 30 mature cheese), and milk (*n* = 30 pasteurized milk and *n* = 30 raw milk).

The samples were placed individually in sterile bags, labeled, and stored in a cooler for cold transport to the laboratory.

### 2.2. Isolation and Identification of Listeria monocytogenes

Each sample of 25 g was mixed in 225 mL of peptone water (Becton Dickson & Co., Cuautitlán Izcalli, Mexico) to obtain a 1:9 proportion and incubated for 24 h at 37 °C. After incubation, plates with CHROMagar™ *Listeria* (CHROMagar Company, Paris, France) were inoculated and incubated at 37 °C for 24 h. Consider a typical appearance of the *L. monocytogenes* colony: blue in color, diameter less than 3 mm, and regular white halo (manufacturer’s manual).

Identification was made via MALDI-TOF mass spectrophotometry and polymerase chain reaction (PCR), using *L. monocytogenes* CCM5577 as a positive control.

The mass spectrum ranged from 2000 to 20,000 Da, and it was generated with the VITEK MS Plus mass spectrometer (bioMerieux, Marcy l’Etoile, France). For each bacterial sample, mass fingerprints were processed by Compute Engine and the advanced spectrum classifier (ASC) algorithm of the VITEK MS system, which automatically identifies a species by comparing the obtained spectrum (presence or absence of specific peaks) with the spectra typical of each claimed species (VITEK MS IVD version 3.0.0). In line with the manufacturer’s instructions, a confidence interval of 60–99% was considered acceptable for species level identification (ID).

For species identification by PCR, bacterial DNA extraction is first obtained from a pure culture on tryptic soy agar (BD Becton Dickinson & Co., Cuautitlán Izcalli, Mexico) by lysis of a bacterial cell suspension at 95 °C for 15 min, followed by centrifugation at 13,000× *g* for 3 min [24].

A PCR was performed to identify the isolates as *L. monocytogenes* by using the amplification of gene *lmo*224 at the 420 base pair (bp) (F-TGTCCAGTTCCATTTTTAACT and R-TTGTTGTTCTGCTGTACGA) [25]. The reaction mixture contained 5× buffer (5X Colorless GoTaq^®^ Flexi Buffer, Promega, Madison, WI, USA), 25 mM MgCl_2_ (Magnesium Chloride Solution, 25 mM, Promega, USA), 10 µM dNTPs (Bioline, Taunton, MA, USA), 10 µM primers, 5 U/µL Taq DNA polymerase (Promega, USA), and sterile water in a final volume of 25 mL.

Amplification conditions were as follows: denaturation at 94 °C for 3 min, followed by 35 cycles of 94 °C for 40 s, 52 °C for 1.15 min, and 72 °C for 1.15 min, and finally one cycle at 72 °C for 7 min in a thermocycler Veriti^TM^ 60 well Thermal Cycler (Applied Biosystems^TM^, Waltham, MA, USA). PCR products were evaluated in 2.5% agarose gels with TBE 0.5X at 1.5% (*w/v*), with SYBR Gold (Invitrogen, Paisley, UK) and molecular marker (100 pb Promega, WI, USA) at 100 V for 45 min.

### 2.3. Classification of L. monocytogenes Serogroups

Strains confirmed as *L. monocytogenes* were further analyzed for serogroup identification by PCR, as described by Doumith et al. [26]. The serotypes of *L. monocytogenes* are grouped into four PCR serogroups:

Serogroup 1—Comprising strains of serovars 1/2a and 3a (amplification of only the *lmo0*737 DNA fragment).

Serogroup 2—Comprising strains of serovars 1/2c and 3c (amplification of both the *lmo0*737 and *lmo*1118 DNA fragments).

Serogroup 3—Comprising strains of serovars 1/2b, 3b, and 7 (amplification of only an ORF2819 DNA fragment).

Serogroup 4—Comprising strains of serovars 4b, 4d, and 4e (amplification of both ORF2819 and ORF2110 DNA fragments).

The marker genes selected for the multiplex PCR assay were *lmo*0737 and *lmo*1118, identified in *L. monocytogenes* serovar 1/2a, and ORF2819 and ORF2110, identified in *L. monocytogenes* 4b. The *prs* gene, specific for strains of the genus *Listeria*, was targeted for an internal amplification control (Table 1) [26].

The PCR mixture contained 5X buffer, 25 mM MgCl_2_, 10 mM dNTPs, 10 mM of each primer, and 5 U Taq DNA polymerase. The PCR amplification conditions were as follows: initial denaturation at 95 °C for 1 min, followed by 30 cycles of denaturation at 95 °C for 45 s, annealing at 53 °C for 45 s, extension at 72 °C for 45 s, and a final cycle of amplification at 72 °C for 7 min. Verification of the PCR products was performed using 2% agarose gel at 100 V for 45 min. *L. monocytogenes* 4b ATCC 13932 was used as positive control, and nuclease-free water was used as negative control.

The classification of the serogroups was carried out as described by the protocol of Doumith et al. [26], considering the presence/absence of the genes (Table 2).

### 2.4. Virulence Factors

PCR was carried out using target gene-specific primers, including those for genes encoded in LIPI-1 (*ac*tA, *hly*, *mpl*, *plcA*, *plcB* and *prfA*), LIPI-2 (*inlA*, *inlB* and *inlC*), and LIPI-3 (*llsX*) (Table 3) [27,28,29,30,31,32,33].

PCR mix was prepared in a total reaction volume of 20 μL, as follows: buffer 5X, MgCl_2_ 25 mM, dNTPs 10 mM, primer 10 mM, Taq DNA polymerase 5 U. The PCR cycling conditions were as follows: the initial denaturation step at 1 min at 95 °C, 30 cycles of 95 °C for 45 s, variable alignment temperature depending on the primers (Table 3) for 45 s, 72 °C for 45 s, and a final extension at 72 °C for 7 min.

Verification of PCR products was performed in electrophoresis using 2% agarose gel.

### 2.5. Antimicrobial Susceptibility Testing

This test was performed according to the Clinical & Laboratory Standards Institute (CLSI) and the European Committee on Antimicrobial Susceptibility Testing (EUCAST) manual [34,35], by using the Kirby–Bauer method. The strains at a concentration of 0.5 McFarland were inoculated on a Mueller–Hinton agar plate (Becton Dickinson & Co., Franklin Lakes, NJ, USA). The antimicrobial disks were individually firmly placed on the inoculated plate. The plates were incubated at 37 °C for 24 h. The antimicrobials tested in this study were ampicillin (AM; 10 µg), chloramphenicol (C; 30 µg), ciprofloxacin (CIP; 5 µg), erythromycin (E; 15 µg), gentamicin (GM; 10 µg), levofloxacin (LEV; 5 µg), meropenem (MEM; 10 µg), penicillin (P; 10 µg), streptomycin (STR; 10 µg), sulfamethoxazole/trimethoprim (STX/TMP; 23.75/1.15 µg), tetracycline (TE; 30 µg), and vancomycin (VAN; 30 µg) (Sensi-Disk^TM^, Becton Dickson & Co., USA). After incubation, the diameter of the clear zone of inhibition around each antimicrobial disk was measured in millimeters and the results were interpreted according to interpretative criteria provided by the CLSI and EUCAST.

A multiple antibiotic resistance (MAR) index was determined by using the number of antibiotics to which each isolate was resistant, along with the total number of antibiotics that were evaluated. Results over or equal to 0.2 indicate an intensive and inappropriate use of resistant antibiotics and represent a high risk of promoting antibiotic resistance [36,37].

### 2.6. Statistical Analysis

Analysis of the positive and negative samples was carried out with the chi-square test in the GraphPad Prism version 5.03 software.

A matrix was constructed using the data belonging to the virulence factors, which were evaluated in a dichotomic fashion, where 1 represented the presence of a gene and 0 represented its absence. As for the antimicrobials, resistance was represented with 1, the intermediate with 0.5, and susceptibility with 0. This matrix was used for the construction of the heatmap and the correlation matrix, both performed in R Studio [38]. A heatmap graph was plotted with the package “ComplexHeatmap” [39]. The optimal number of clusters in the dendrograms used in the heatmap was calculated with the package “factoextra” with the function “fviz_nbclust” and the “wss” and “silhouette” methods. The correlation matrix was constructed with the “metan” package with the functions “corr_coef” and “plot”, using Spearman’s correlation [40].

## 3. Results

### 3.1. Sample Collection

A total of 300 samples were collected.

### 3.2. Isolation and Identification of Listeria monocytogenes

A total of 300 samples were analyzed for *L. monocytogenes*, of which 5.6% (17/300) were positive to *L. monocytogenes* according to the specific characteristics of CHROMagar™ *Listeria.* Positive samples were only identified in ground beef, chicken, and cheese; in the rest of the foods (seafood and milk), it was not present. In the ground beef, 10% (6/60) positive samples were detected; for chicken, this was only 5% (3/60), and for fresh cheese, it was 26.6% (8/30). From each positive sample, one to five strains were isolated and identified as *L. monocytogenes*, making a total of 66 strains. All strains were analyzed by PCR, considering only those that presented the lmo224 gene positive for *L. monocytogenes*. To confirm the identification of the species, the strains were also analyzed using MALDI-TOF, considering only those that presented a confidence interval greater than 98% for the identification of *L. monocytogenes* as positive. Afterward, tests were carried out to rule out possible clones, leaving only 38 strains in the end (11 ground beef strains, 4 chicken strains, and 23 cheese strains).

### 3.3. Classification of Serogroups

Three out of four possible PCR serogroups were identified in the 38 evaluated strains. Most of the strains were serogroup 4b, 4d, 4e (68.4%; 26/38), followed by 1/2a, 3a (23.7%; 9/38) and 1/2b, 3b (7.9%; 3/38). No strains were detected for serogroup 1/2c, 3c (0.0%; 0/38) (Table 4).

### 3.4. Virulence Factors

Of the ten virulence factors evaluated, all strains (38/38) showed a minimum of 4 and a maximum of 8. None of the assessed strains harbored all virulence genes.

In 21.0% (8/38) of the strains, four virulence factors were detected; in 7.8% (3/38), there were five; in 23.6% (9/38), six were detected; in 18.4% (7/38), there were seven; and in 28.9% (11/38), eight virulence factors were identified. The genes found most frequently in the strains were *actA*, *hly,* and *plc*B, at 92.1% (35/38). On the contrary, the virulence factors with the least frequency were *mpl* and *prf*A, in 21.0% (8/38) of the strains (Table 5).

### 3.5. Antimicrobial Susceptibility Test

In total, 78.9% (30/38) of the strains showed resistance to at least one antibiotic tested, and 21.0% (8/38) were susceptible to all antibiotics. All strains (38/38) were susceptible to AM, C, GM, LEV, P, TE, and VAN. The strains were mostly resistant to STX-TMP, at 78.9% (30/38); STR, at 26.3% (10/38); MEM, at 21.0% (8/38); and E, at 2.6% (1/38) (Table 6). Multidrug resistance was detected in 13.1% (5/38) of the strains, showing resistance to at least three different antimicrobials from different classes.

The multiple antibiotic resistance index (MARI) values ranged from 0.083 to 0.250. Only 13.1% (5/38) of the strains analyzed had values greater than 0.2 (Table 7).

**Figure 1:**
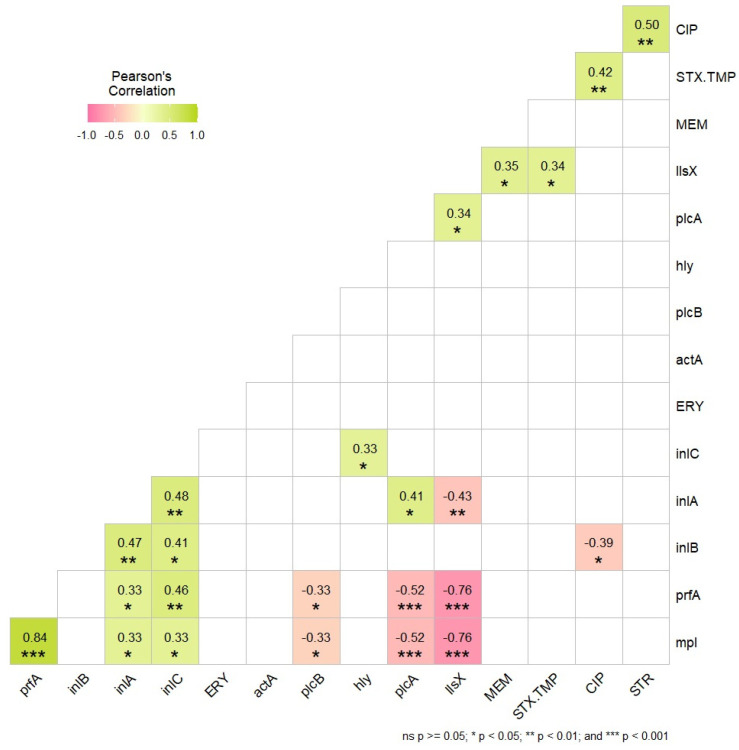
Correlation matrix of virulence factors (*n* = 10) and only the four antibiotics to which the strains showed resistance. Values indicating significant correlation (*p* < 0.05) are visualized. (Virulence factors: *prf*A, *inl*A, *inl*B, *inl*C, *act*A, *plc*B, *hly*, *plc*A; antibiotics: ERY—erythromycin, MEM—meropenem, STX-TMP—sulfamethoxazole/trimethoprim, CIP—ciprofloxacin, STR—streptomycin).

The results of the correlation analysis of the virulence factors revealed a strong positive correlation between the *lls*X and *prf*A genes (−0.76 ***), the *lls*X and *mpl* genes (−0.76 ***), and the *plc*A and *prf*A genes (−0.52 ***), thus also the *plc*A and *mpl* genes (−0.52 ***) (Figure 1). A moderate positive correlation between the *lls*X and *inl*A genes (−0.43 **) was obtained.

**Figure 2:**
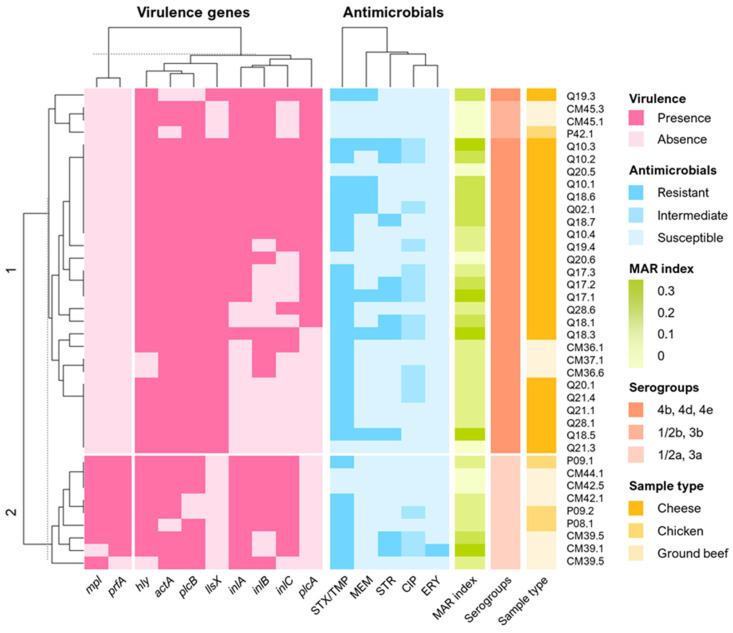
Clustering analysis relation of *L. monocytogenes* strains isolated in this study. The presence or absence of virulence factors and resistance or susceptibility of antimicrobials are shown in dichotomic values. Additionally, scales of MARIs, serotypes, and sample types are shown.

The heatmap in Figure 2 shows the presence or absence of the virulence factors. We found two main clusters, in which 29 strains belonged to cluster 1, and 9 to cluster 2; along with this, 16 subclusters were identified. Clade 1 englobes all strains of serogroups 4b, 4d, 4e and 1/2b, 3b; interestingly, all belong to lineage I. All nine strains in clade 2 were represented by serogroup 1/2a, 3a from lineage II. We could not observe the presence of the *mpl* and *prf*A genes in clade 1, but that was not the case for gene *lls*X, which was only present in the strains of lineage I. This is corroborated by the findings of Figure 1, where these genes exhibit a higher negative correlation, signifying their absence relative to other genes.

## 4. Discussion

*L. monocytogenes* is the bacterium responsible for a foodborne disease named listeriosis, which is not mandatory to be reported by national authorities in Mexico. The absence of official reports about this disease in that country has led to an undetermined potential risk to public health via the consumption of diverse foods that may be contaminated.

In this study, we found an overall prevalence of 5.6% (17/300) in different raw foods from Reynosa, Tamaulipas, Mexico. No strains of *L. monocytogenes* were detected in the seafood and milk (raw or pasteurized) samples included in this study. However, despite these results, it is important to note that its presence has been reported in a range of 0.5 to 15.5% in studies carried out in other countries [41,42,43,44,45]. The fact that *L. monocytogenes* was absent in the raw milk (*n* = 30) and pasteurized milk (*n* = 30) samples might seem like an expected result since pasteurized milk should prevent bacteria growth. However, several authors have reported the presence of *L. monocytogenes* in pasteurized milk, showing an average prevalence of 12.5% (0.1–20.0%) [46,47,48], while in the same studies, for raw milk, the prevalence of *L. monocytogenes* was 4.9% (2.0–7.6%) [46,47,48]. This led us to assume that the presence of *L. monocytogenes* in pasteurized milk could be due to a deficient process or poor hygienic handling practices. For Mexico, Ríos-Muñiz et al. [49] and Silva et al. [50] have described the absence of *L. monocytogenes* strains in raw milk (pasteurized milk samples were not included in these studies).

Among the food samples positive for *L. monocytogenes*, fresh cheese showed a prevalence of 26.6% (8/30), but the bacteria were absent in all the mature cheeses (0/30). This level of prevalence in fresh cheeses (26.6%) might seem high compared with what has been published for various countries, at less than 9.3% [51,52,53,54,55,56,57], and a few studies have reported a higher prevalence range of 17.6 to 60% [45,47,58]. The variation in the prevalence of *L. monocytogenes* may be due to several factors. For example, the raw or pasteurized milk with which cheeses are prepared can be contaminated due to inadequate pasteurization or post-pasteurization contamination [59]. Furthermore, it should be considered that contamination can occur at different stages during cheese production since processing plants can harbor *L. monocytogenes* in the environment and on equipment [60].

Raw chicken samples are known to be one of the main foods susceptible to *L. monocytogenes* contamination [56,57]. This study had a prevalence of 5% (3/60), similar to the 3.5% reported by Zhang et al. in China [61]. However, this can be considered a low percentage compared with the results obtained in other similar studies, of 8.5 to 53.3% [57,62,63,64,65,66,67]. In contrast to the findings reported by Mamber et al. [68], our results showed a higher percentage. In their study, 0.46% of chicken samples collected from the USA were tested positive for *L. monocytogenes.* It is important to consider that *L. monocytogenes* contamination of poultry meat may occur during production, processing, and storage [62,65]. Therefore, good thermic treatment, personnel hygiene, cleaning applications, and processing and storage conditions may prevent *L. monocytogenes* contamination during food processing [65,67].

The ground beef samples included in the current study had an *L. monocytogenes* prevalence of 10% (6/60). These results are similar to those reported in other studies, like 7.3% in South Africa [69], 8.9% in India [64], 9.0% in the United States [70], 9.1% in China [71], 14% in Brazil [72], and 14% in Egypt [44]. According to these comparisons, beef appears to be treated in the same way and with the same hygiene handling processes. Although the percentage of *L. monocytogenes* in the samples seems low, its presence still represents a risk to the consumer’s health. Therefore, it is necessary to improve meat handling practices throughout the production chain, as well as how to handle the meat once purchased, avoid cross-contamination in the kitchen, and ensure correct cooking.

From the 38 strains positive for *L. monocytogenes* in the current study, three of the four possible PCR serogroups were identified (present: serogroup 1/2a, 3a, serogroup 1/2b, 3c and serogroup 4b, 4d, 4c; absent: serogroup 1/2c, 3c). The multiplex PCR profiles did not distinguish serovar 1/2a from 3a, 1/2c from 3b, and 7 or 4b from 4d and 4e within *L. monocytogenes*. However, serovars 3a, 3b, 3c, 4a, 4c, 4e, 4d, and 7 are very infrequent in food and rarely reported as implicated in human listeriosis [73].

Serotypes 4b, 1/2a, and 1/2b have been related to listeriosis cases in humans and are responsible for 95% of the cases reported worldwide [74,75,76]. Still, among these serotypes, 4b stands out for being associated with more than half of human clinical cases, while strains of serotypes 1/2a or 1/2b have only been associated with sporadic cases of listeriosis in Europe and North America [73,74,76,77,78]. In previous studies, serotype 1/2a has been predominant while serotype 4b has usually been underrepresented [73,74,75]. In the current results, serotype 4b was dominant in foods. Locatelli et al. [75] have suggested that strain competition among *L. monocytogenes* serotypes 1/2a, 1/2b, 1/2c, and 4b is merely the result of bacterial interactions on the meat product among strains of the same species with almost the same growth potential.

These serotypes are further bifurcated into four major genetic diversity lineages: lineage I, lineage II, lineage III, and lineage IV. Each lineage possesses specific serotypes. The strains included in the current study were classified into only two lineages: serotypes 4b, 4d, 4c and 1/2b, 3b within lineage I and serotype 1/2a, 3a in lineage II. None of the strains was identified as lineage III or IV. Serotypes 1/2b and 4b within lineage I are encoded for the virulence factor called listeriolysin S, which is not found in other lineages [8,73]. Lineage II harbors 1/2a, 3a, which has numerous plasmids that are resistant to heavy metals and antibiotics [8]. Most strains isolated in this study exhibited serotypes associated with listeriosis (4b and 1/2a = 92.1%, 35/38), which can represent a potential risk to public health.

The ability of *L. monocytogenes* to infect a human host and cross the intestinal barrier, reaching other pivotal parts of the body, is highly related to the presence of pathogenic islands (LIPI-1, LIPI-3 and LIPI-4) [79,80]. Current investigations indicate that virulence factors are key for the adaptation of *L. monocytogenes* to its host and its optimal spread in the environment [81]. Internalin A is a major factor in inducing the internalization of *L. monocytogenes* in epithelial cells, and internalin B is important in placental invasion [80]. For this use, two internalins, A (*Inl*A) and B (*Inl*B), present in the genome, with a minor contribution by the toxin listeriolysin O (LLO), induce the uptake of the bacterium [82]. In this study, a moderate correlation was obtained between *inl*A and *lls*X (0.43). The internalins *inl*A, *inl*B, and *inl*C were found at 71.1% (27/38), 63.2% (24/38), and 55.3% (21/38), respectively. The presence of these internalins indicates the ability of these strains to cause listeriosis.

For its part, *prf*A prepares *L. monocytogenes* for internalization and intracellular life, inducing the transcription of LIPI-1, the main virulence regulon [82]. LIPI-1 includes *prf*A itself and genes encoding listeriolysin O (*hly*), phospholipase A (*plc*A), phospholipase B (*plcB*), actin assembly inducing-protein (*actA*), a zinc metalloproteinase (*mpl*), and *orf*X (*orfX*) [80]. This explains the correlation existing in the current results between the virulence factors *prf*A and *plc*A (0.52), as well as between *plc*A and *mpl* (0.52). The presence of *prf*A in these strains tells us their ability to form biofilms that may provide selective pressure to maintain this critical virulence regulation when *L. monocytogenes* is outside of host cells in the environment.

*L. monocytogenes* infections are commonly treated with antibiotics such as ampicillin, gentamicin, penicillin, trimethoprim/sulfamethoxazole, vancomycin, and erythromycin [4,8,9,10]. The most common antibiotic regimen prescribed for listeriosis includes penicillin/ampicillin alone or in combination with aminoglycosides (gentamicin) [70]. In our results, the *L. monocytogenes* strains analyzed were shown to be 100% sensitive to ampicillin and 97.2% to gentamicin, indicating that these continue to be effective treatments.

The strains isolated in this study were tested with a panel of 12 antibiotics, showing resistance to only 4 of these: sulfamethoxazole/trimethoprim, at 78.9%; streptomycin, at 26.3%; meropenem, at 21.1%; and gentamycin, at 2.6%. Most of the strains were resistant to sulfamethoxazole/trimethoprim (78.9%), which serves as a critical warning, as sulfamethoxazole/trimethoprim is an alternative or secondary treatment for listeriosis in meningoencephalitis caused by *L. monocytogenes* [83,84]. Except for the sulfamethoxazole/trimethoprim combination (78.9%), which showed high resistance against *L. monocytogenes* isolates, the strains showed high susceptibility to most of the antibiotics tested, coinciding with the susceptibility pattern commonly reported in other studies.

## 5. Conclusions

The results obtained in this study revealed a low prevalence of *L. monocytogenes* in foods marketed in Reynosa, Tamaulipas. The bacteria were identified only in fresh cheese, beef, and chicken, with mainly the strains of serogroup 4b, 4d, 4e associated with listeriosis. All strains presented at least four virulence genes; the most common were *actA*, *hly,* and *plcB*, which indicates their pathogenic capacity and potential risk for consumers. The strains exhibited a high percentage of antimicrobial susceptibility, showing that common treatments remain effective in most cases. However, the resistance observed relative to STX-TMP and STR deserves attention as to whether it will continue to increase until it represents a problem of ineffectiveness in the treatments. The need to improve measures against policies regarding and surveillance of *L. monocytogenes* in Reynosa, Tamaulipas, is emphasized. Likewise, the monitoring of antibiotic susceptibility patterns is recommended, considering that a change in these patterns could occur depending on management practices in the productive chain.

## Figures and Tables

**Table 1:** Primers used in the study and their sequences.

Primer’s Name	Primers Sequences (5’→3’)	Product Size (pb)
*lmo0737*	AGGGCTTCAAGGACTTACCC ACGATTTCTGCTTGCCATTC	691
*lmo1118*	AGGGGTCTTAAATCCTGGAA CGGCTTGTTCGGCATACTTA	906
ORF2819	AGCAAAATGCCAAAACTCGT CATCACTAAAGCCTCCCATTG	471
ORF2110	AGTGGACAATTGATTGGTGAA CATCCATCCCTTACTTTGGAC	597
*prs*	GCTGAAGAGATTGCGAAAGAAG CAAAGAAACCTTGGATTTGCGG	370

**Table 2:** Interpretation of results of amplified genes for the identification of molecular serogroups.

Gene	bp	Serogroups								
		1/2a	3a	1/2c	3c	4b	4d	4e	1/2b	3b
*lmo1118*	906			✓	✓					
*lmo0737*	691	✓	✓	✓	✓					
ORF2110	597					✓	✓	✓		
ORF2819	471					✓	✓	✓	✓	✓
*prs*	370	✓	✓	✓	✓	✓	✓	✓	✓	✓

**Table 3:** Primers used for detection of virulence genes of *L. monocytogenes*.

Prime’s Name	Primers Sequences (5’→3’)	T/A (°C)
*actA*	CGCCGCGGAAATTAAAAAAAGA ACGAAGGAACCGGGCTGCTAG	60
*hly*	GTTAATGAACCTACAAGACCTTCC ACCGTTCTCCACCATTCCCA	60
*llsX*	TTATTGCATCAATTGTTCTA CCCCTATAAACATCATGCTAGTG	52
*mpl*	GCTTTGCCGGATTCCTGCG CTTCTTATTCGCCCATCTCGCG	55
*plcA*	CTGCTTGAGCGTTCATGTCTCATCCCCC CATGGGTTTCACTCTCCTTCTAC	60
*plcB*	ATGTGCTTGACCGCAAGTGT CTTCTCGGTAATCAGCCACC	60
*prfA*	AACGGGATAAAACCAAAACCA TGCGATGCCACTTGAATATC	60
*inlA*	CGGATGCAGGAGAAAATCC CTTTCACACTATCCTCTCC	60
*inlB*	GATATTGTGCCACTTTCAGGT CCTCTTTCAGTGGTTGGGT	60
*inlC*	AATTCCCACAGGACACAACC CGGGAATGCAATTTTTCACTA	55

**Table 4:** PCR serogroups for *L. monocytogenes* strains per sample type.

Type of Sample		% (*n*)	
	4b, 4d, 4e	1/2b, 3b	1/2a, 3a
Cheese (*n* = 23)	100% (23/23)	0.0% (0/23)	0.0% (0/23)
Chicken (*n* = 4)	0.0% (0/4)	25.0% (1/4)	75.0% (3/4)
Ground beef (*n* = 11)	27.3% (3/11)	18.2% (2/11)	54.5% (6/11)

**Table 5:** Prevalence of virulence factors in *L. monocytogenes* strains.

Pathogenicity Islands	Virulence Factors	Serogroups		
		1/2a, 3a	1/2b, 3b	4b, 4d, 4e
LIPI-1	*act*A	88.8% (8/9)	66.6% (2/3)	96.1% (25/26)
	*hly*	88.8% (8/9)	100.0% (3/3)	92.3% (24/26)
	*mpl*	88.8% (8/9)	0.0% (0/3)	0.0% (0/26)
	*plc*A	0.0% (0/9)	100.0% (3/3)	61.5% (16/26)
	*plc*B	77.7% (7/9)	100.0% (3/3)	96.1% (25/26)
	*prf*A	88.8% (8/9)	0.0% (0/3)	0.0% (0/26)
LIPI-2	*inl*A	100.0 (9/9)	100.0% (3/3)	57.6% (15/26)
	*inl*B	77.7% (7/9)	100.0% (3/3)	53.8% (14/26)
	*inl*C	88.8% (8/9)	0.0% (0/3)	50.0% (13/26)
LIPI-3	*lls*X	0.0% (0/9)	0.0% (0/3)	100.0% (26/26)

**Table 6:** Antimicrobial susceptibility test results of *L. monocytogenes* strains.

Antimicrobials	Lineage I						Lineage II		
	4b, 4d, 4e			1/2b, 3b			1/2a, 3a		
	S	I	R	S	I	R	S	I	R
Ampicillin	100% (26/26)	0.0% (0/26)	0.0% (0/26)	100% (3/3)	0.0% (0/3)	0.0% (0/3)	100% (9/9)	0.0% (0/9)	0.0% (0/9)
Chloramphenicol	100% (26/26)	0.0% (0/26)	0.0% (0/26)	100% (3/3)	0.0% (0/3)	0.0% (0/3)	100% (9/9)	0.0% (0/9)	0.0% (0/9)
Ciprofloxacin	53.8% (14/26)	46.1% (12/26)	0.0% (0/26)	100% (3/3)	0.0% (0/3)	0.0% (0/3)	66.6% (6/9)	33.3% (3/9)	0.0% (0/9)
Erythromycin	100% (26/26)	0.0% (0/26)	0.0% (0/26)	100% (3/3)	0.0% (0/3)	0.0% (0/3)	88.8% (8/9)	0.0 (0/9)	11.1% (8/9)
Gentamicin	100% (26/26)	0.0% (0/26)	0.0% (0/26)	100% (3/3)	0.0% (0/3)	0.0% (0/3)	100% (9/9)	0.0% (0/9)	0.0% (0/9)
Levofloxacin	100% (26/26)	0.0% (0/26)	0.0% (0/26)	100% (3/3)	0.0% (0/3)	0.0% (0/3)	100% (9/9)	0.0% (0/9)	0.0% (0/9)
Meropenem	69.7% (20/26)	0.0% (0/26)	30.7% (8/26)	100% (3/3)	0.0% (0/3)	0.0% (0/3)	100% (9/9)	0.0% (0/9)	0.0% (0/9)
Penicillin	100% (26/26)	0.0% (0/26)	0.0% (0/26)	100% (3/3)	0.0% (0/3)	0.0% (0/3)	100% (9/9)	0.0% (0/9)	0.0% (0/9)
Streptomycin	69.7% (20/26)	0.0% (0/26)	30.7% (8/26)	100% (3/3)	0.0% (0/3)	0.0% (0/3)	77.7% (7/9)	0.0% (0/9)	22.2% (2/9)
Sulfamethoxazole/trimethoprim	11.5% (3/26)	0.0% (0/26)	88.4% (3/26)	100% (3/3)	0.0% (0/3)	0.0% (0/3)	22.2% (2/9)	0.0% (0/9)	77.7% (7/9)
Tetracycline	100% (26/26)	0.0% (0/26)	0.0% (0/26)	100% (3/3)	0.0% (0/3)	0.0% (0/3)	100% (9/9)	0.0% (0/9)	0.0% (0/9)
Vancomycin	100% (26/26)	0.0% (0/26)	0.0% (0/26)	100% (3/3)	0.0% (0/3)	0.0% (0/3)	100% (9/9)	0.0% (0/9)	0.0% (0/9)

S = susceptible, I = intermediate, R = resistant.

**Table 7:** Multiple antibiotic resistance indexes (MARIs) of *L. monocytogenes* strains.

MARI	Pattern	*n*	Total	%
0.083	STX-TMP	16	16/38	42.1
0.167	STX-TMP + STR STX-TMP + MEM	5 4	9/38	23.6
0.250	STX-TMP + STR + MEM STX-TMP + STR + ERY	4 1	5/38	13.1

## Data Availability

The original contributions presented in the study are included in the article, further inquiries can be directed to the corresponding author.
